# No evidence of neuronal/glial autoantibodies in febrile infection-related epilepsy syndrome (FIRES): a prospective clinic-serologic analysis

**DOI:** 10.3389/fnins.2023.1221761

**Published:** 2023-07-13

**Authors:** Janina Soler Wenglein, Gerhard Kluger, Frank Leypoldt, Klaus-Peter Wandinger, Andreas van Baalen

**Affiliations:** ^1^Department of Neuropediatrics, University Medical Center Schleswig-Holstein, Kiel University (CAU), Kiel, Germany; ^2^Clinic for Neuropediatrics and Neurorehabilitation, Epilepsy Center for Children and Adolescents, Schön Clinic Vogtareuth, Vogtareuth, Germany; ^3^Research Institute for Rehabilitation, Transition, and Palliation, Paracelsus Medical University Salzburg, Salzburg, Austria; ^4^Department of Neurology, University Medical Center Schleswig-Holstein, Kiel University (CAU), Kiel, Germany; ^5^Neuroimmunology Section, Institute of Clinical Chemistry University Medical Center Schleswig-Holstein, Kiel, Germany

**Keywords:** FIRES, NORSE, status epilepticus, encephalopathy, autoimmune, inflammation, AE, autoantibodies

## Abstract

The pediatric febrile infection-related epilepsy syndrome (FIRES) manifests with encephalopathy with super-refractory status epilepticus (SE) a few days after or accompanying a febrile illness. It often results in refractory epilepsy and cognitive dysfunction in previously healthy children and adolescents. The underlying pathomechanism is unknown, which is why causative neuronal and/or synaptic antibodies have been discussed. We report a prospective consecutive cohort of 14 children (10 male, four female) diagnosed with FIRES in the acute phase, whose serum and CSF were comprehensively screened for underlying synaptic/neuronal autoantibodies. The median age at onset was 6  years (range 4–9  years). None of the children had a medical history of epilepsy. Duration of SE varied from less than 1 week to 2.5  months (Median: 1  month, range  < 1  week-2.5  months). Clinical response to treatment with antiseizure medications was poor as well as the outcome: one child died in the acute phase of SE, and two died in the long term. All surviving children showed neuropsychological impairments. No underlying synaptic or neuronal autoantibodies were identified in 13 of 14 children’s sera or CSF. One child had currently uncharacterized neuronal autoantibodies in CSF, yet clinical presentation was atypical for FIRES. Based on our findings, the child was later diagnosed with autoimmune encephalitis (AE). We conclude that FIRES is not an autoantibody-mediated disease. However, a comprehensive screening for known and yet unknown antineuronal antibodies in serum and CSF is warranted to rule out AE mimicking FIRES.

## 1. Introduction

Febrile infection-related epilepsy syndrome (FIRES) describes the sudden onset of an epileptic encephalopathy, starting with a therapy-refractory status epilepticus (SE) in previously healthy children. It typically runs in two phases: an initial (usually mild) febrile infection without cerebral involvement, followed by a devastating SE. Typically in FIRES, there are no significant findings in the cerebrospinal fluid (CSF). CSF is normal, at most with a subtle pleocytosis, normal protein concentrations, and there are no oligoclonal bands (Caraballo et al., 2013). After the acute phase, patients develop chronic epilepsy and often global brain atrophy accompanied by moderate to severe neuropsychological limitations (Gaspard et al., 2018). Since FIRES follows or begins with a febrile illness and there is no evidence of an infectious agent causing the clinical picture, a post-infectious inflammatory, possibly antibody-mediated pathomechanism has repeatedly been suspected (Caraballo et al., 2013; van Baalen et al., 2017). Previous research addressed genetic causes, metabolic diseases, infectious and inflammatory processes, but none lead to a proven etiology of FIRES (Appenzeller et al., 2012; van Baalen et al., 2017; Lee and Chi, 2018).

Recently, pediatric autoimmune encephalitis (AE) has increasingly been recognized to underlie some cases of new-onset status epilepticus (Armangue et al., 2014) with autoantibodies targeting neuronal [NMDA receptor, GABA(A) receptor, GAD65] and glial autoantigens (Myelin oligodendrocytic glycoprotein antibody associated diseases MOGAD) (Leypoldt et al., 2015; Dalmau and Graus, 2018; Armangue et al., 2020; Banwell et al., 2023). In some of these cases, prodromal infection-like syndromes in anti-NMDAR encephalitis (Dalmau et al., 2011) or relapsing febrile episodes in MOGAD have a strong phenotypic overlap with FIRES (Udani et al., 2021). Indeed, there have been previous case reports describing AE in patients initially suspected to have FIRES (Caputo et al., 2018).

In a previous study, we could not identify underlying antineuronal antibodies in a retrospective cohort of 12 children with FIRES. Though, at that time, only a few sera were obtained during the acute phase, and in most cases, CSF was not available (van Baalen et al., 2012). Therefore, the aim of this study was to comprehensively analyze neuronal and glial autoantibodies in serum and CSF, obtained early during the disease in a prospective cohort of children with suspected FIRES.

## 2. Methods

Between 2014 and 2018, we prospectively recruited 14 children with suspected FIRES seen at 14 university-based pediatric neurology departments. Inclusion criteria were: (1) Status epilepticus (SE) following a febrile illness without history of a previous epilepsy, (2) no evidence of other causes, and (3) cerebral spinal fluid (CSF) available. In addition, a serum sample from the acute phase (within 4 weeks of onset) must have been available. Children were characterized as typical and atypical FIRES by authors (JSW and AvB). Samples left from routine serum and CSF analysis were comprehensively screened for synaptic and neuronal autoantibodies using a comprehensive rat-brain tissue-based screening and cell-based assay confirmation approach developed by Dalmau and colleagues (Dalmau et al., 2008, 2011; Dalmau and Vincent, 2017; Wandinger et al., 2018).

In brief, all samples were screened via tissue-based immunohistochemistry (IHC) using sagittal rat brain sections (initial dilution 1:200 serum, 1:4 CSF). Samples that tested positive were examined for known antineuronal antibodies using (1) indirect immunofluorescence on transfected HEK293T cells [NMDA-R NR1, IGLON5, CASPR2, GABAB-R, GABA(A)R, glycine receptor, alpha-neurexin, mGluR5, DPPX, AMPA-R, and LGI1-R] and (2) non-permeabilized embryonal, rat, primary culture neurons. Samples 2–14 were also screened by indirect immunofluorescence for MOG-antibodies using non-permeabilized HEK293T cells, transfected with full-length human MOG vectors as previously described (Titulaer et al., 2014). Screening was performed at the Institute of Clinical Chemistry, University Medical Center Schleswig Holstein, Kiel/Lübeck.

For 11 children (eight male, three female), sufficient information on the medication was available. Treatment data were analyzed regarding drug therapy in the SE from day 1 to 28 (in one case from day 1 to 14, due to death).

The ethics committee of the Faculty of Medicine at the Kiel University (CAU) approved this study (D 414/21), and written informed consent was sought from all parents.

## 3. Results

We recruited 14 children with a median age of 6 years (range 4–9 years); 10 males, four females (Table 1). The causes of the fevers were predominantly simple febrile infections. Eleven children had a recent history of respiratory infection with accompanying general symptoms. Two children previously showed an abdominal infection. One child (#5) had no prodromal phase, no apparent cause of the fever and developed SE on the first day of the fever. Duration of SE ranged from less than a week to 2.5 months (median 1 month). Reported outcomes were poor in all children. One child (#6) died in the acute phase, two children (#7 and #8) died in the long-term, eight children developed a cognitive impairment from mild to severe. For three children (#4, #9, and #10), outcomes were not reported.

**Table 1:** Clinical findings and outcome in 14 children with suspected FIRES.

Child#	Sex (f/m)	Age (y)	Medical history	Fever at onset SE	Duration of SE	Associated clinical and metabolic features	Immunotherapy	Response to immunotherapy	Outcome	Positive proof of antibodies
1	m	5	Negative	No	2–3 weeks	None	Steroid pulse	No	MCI	No
2	f	6	Negative	No	2–3 weeks	Plasma amino acids varying during SE	Immunoglobulins	No	MCI	No
									Visual impairment	
3	m	9	Negative	No	6 weeks	Muscle biopsy abnormal	Steroid pulse	No	MCI	No
							Immunoglobulins	No		
4	f	6	Unknown	Unknown	Unknown	Unknown	Unknown	Unknown	Unknown	No
5	m	6	Pituitary insufficiency with hypothyroidism and hypocortisolism	Yes (first day)	3 weeks	Intrathecal IgM synthesis short duration of illness with SE at first day of fever	Steroid pulse	No	SCI	Unknown Neuronal Antibody
							Steroid substitution	No		
	
6	m	8	Negative	No	2 weeks	Multiple organ failure	Immunoglobulins Steroid pulse	No	Died on day 14	No
7	m	6	Simple febrile seizure at age of 2 years	No	2–3 months	Long and complicated Intensive-care period	Steroid pulse (2x)	No	Minimal responsive state;	No
							Immunoglobulins	No	Died 2 years after SE	
							Anakinra	No		
8	m	7	Negative	No	4 weeks	Clotting disorder	Steroid pulse	No	Minimal responsive state	No
							Immunoglobulins	No	Died 3 years 10 months after SE	
9	m	8	Negative	No	Unknown	Unknown	Unknown	Unknown	Unknown	No
10	m	6	Negative	Yes	2 months	Plasma amino acids varying during SE Interleukin-1b elevated	Immunoglobulins	No	Unknown	No
							Anakinra	No		
	
11	f	4	Negative	No	1 month	Plasma amino acids varying during SE	Steroid pulse	No	SCI	No
							Immunoglobulins	No	Visual impairment	
							Anakinra	No		
12	m	4	Mild developmental retardation	No	Less than 1 week	Mastoiditis without cerebral infection	Steroid pulse	Yes	MCI	No
13	m	7	Negative	No	1–2 months	Plasma amino acids varying during SE	Steroid pulse	No	Minimal responsive state	No
							Long-term therapy with steroids	No		
							Immunoglobulins	No		
							Plasmapheresis	No		
14	f	7	Negative	No	4 weeks	Short period without seizures after first SE	Steroid pulse	No	MCI	No
							Immunoglobulins	No		
							Anakinra (1.5 years after initial SE)	Unknown		

m, male; y, years; f, female; SE, status epilepticus; CSF, cerebrospinal fluid; MCI, mild cognitive impairment; and SCI, severe cognitive impairment.

Cerebrospinal fluid analyses (considered if taken on the first or second day of SE) resulted in leukocyte counts from 0 to 178 cells/μL (median 7 cells/μL), as well as protein levels of 13.2–648 mg/dL (median 35.1 mg/dL). Available data on characteristics of SE showed no (five children) or alternating (five children) awareness and no (two children) or alternating responsiveness (five children) at onset of SE. Seven children showed tonic–clonic and two showed clonic motor phenomena. Five children presented with complex motor phenomena (Table 2). For two children limb movement disorders (#3 and #11) were described. On electroencephalogram (EEG), eight children had focal epileptiform discharges (Table 3). Severe generalized changes were recorded in five children and spike–wave complexes in four children.

**Table 2:** Seizure semiology features (classification based on ILAE glossary).

**Awareness**
Yes = 2
No = 5
Alternating = 5
Unknown = 2
**Responsiveness**
No = 5
Alternating = 2
Unknown = 7
**Cognitive phenomena**
Atonic = 1
Epileptic nystagmus = 2
**Elementary motor phenomena**
Tonic–clonic = 7
Tonic = 2
**Complex motor phenomena**
Automatisms = 3
Hyperkinetic behavior = 2
**Autonomic phenomena**
Respiratory = 1
No data were available for two patients

**Table 3:** Cerebrospinal fluid, EEG classification based on American Clinical Neurophysiology Society’s standardized critical care EEG terminology, imaging and treatment data.

**Cerebrospinal fluid**
CSF pleocytosis > 20/μL on first day of SE
Yes = 1
No = 10
Unknown = 3
CSF pleocytosis >20/μL on second day of SE
Yes = 1
No = 1
Not conducted or unknown = 12
Special findings
Intrathecal IgM synthesis = 1
**EEG**
Seizures
Electroclinical seizure (ECSz) = 1
Electroclinical status epilepticus (ECSE) = 2
Rhythmic and periodic patterns (RRPs)
Generalized (G) = 2
Lateralized (L) = 9
Bilateral independent (BI) = 2
Multifocal (Mf) = 1
Spike-and-wave or sharp-and-wave (SW) = 3
Brief potentially ictal rhythmic discharges = 3
Global slowing = 6
**Imaging during acute phase**
Initially normal = 8
Abnormal MRI
T2/FLAIR hyperintensity = 3
DWI hyperintensity = 6
Unknown = 1
**Treatment**
Antiseizure medication
Benzodiazepines = 11
Non-benzodiazepine antiseizure medication = 11
Continuous infusion during first 28 days of treatment = 11
Unknown = 3
Ketogenic diet = 8
Immunotherapies
Anakinra = 3
IV steroids = 10
IVIG = 9
Plasmapheresis = 1

CSF, cerebrospinal fluid; IV, intravenous; and IVIG, intravenous immunoglobulins.

**Figure 1:**
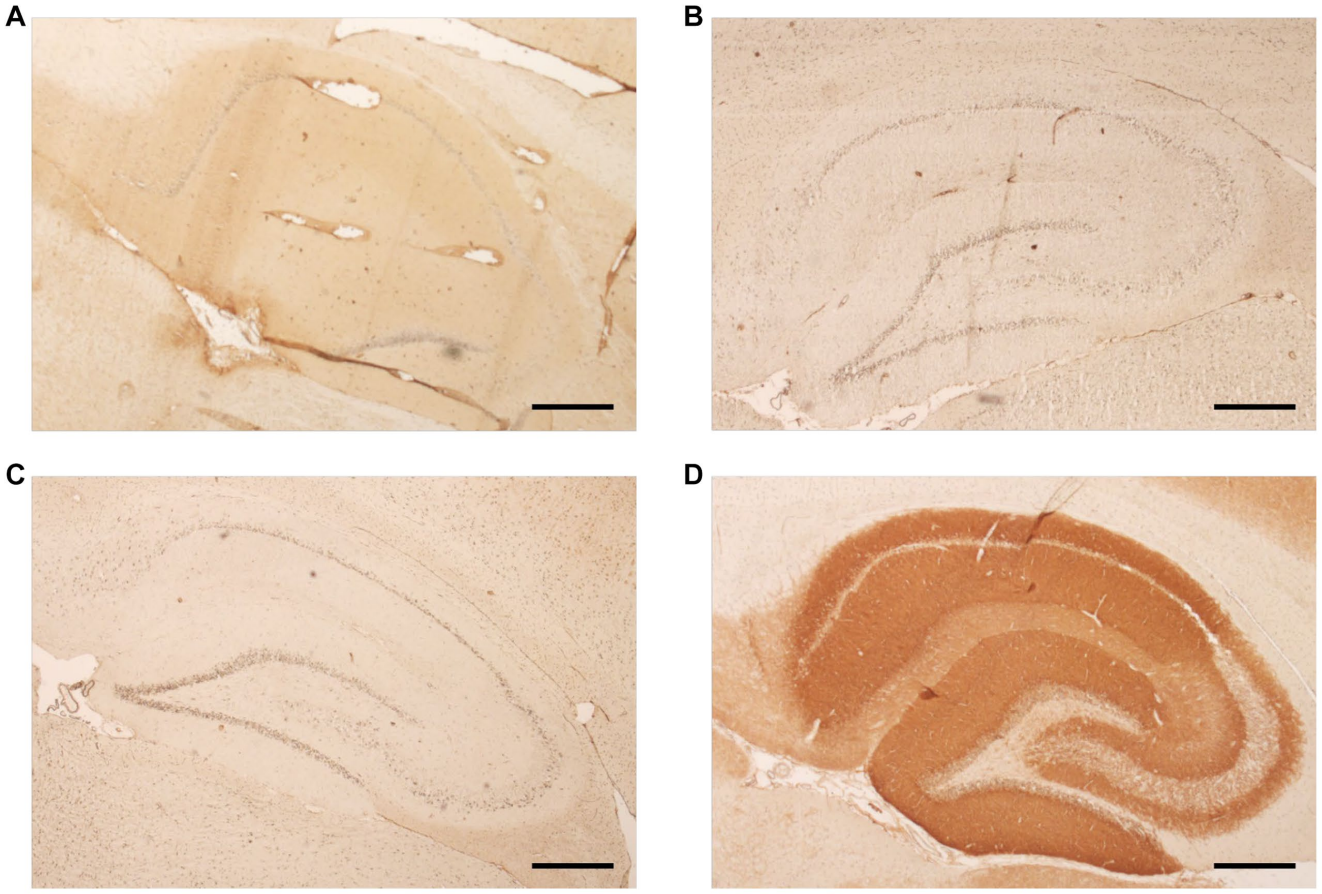
Tissue-based immunohistochemistry (IHC) assay using a specifically prepared, sagittally sectioned rat brain serves as a search and confirmation test. The hippocampal coloration is shown. Panel **(A)** shows a positive result after incubation with CSF (child #5) and proves the presence of an unknown surface antibody. This result was validated by a positive reaction to neuronal surface markers on rat hippocampal primary neuron culture. Subsequent further diagnostic steps to classify the antibody and additional IHC rat brain could not be accessed due to limited CSF volume. Panel **(B)** shows follow-up CSF from the same patient, acquired 5 months after initial sample, it did not show immunoreactivity on rat brain observed by absence of coloration after incubation with CSF. Representative results after incubation with a negative CSF and an NMDA positive CSF are shown in panels **(C, D)**, respectively. Scale bar: 500 μm.

All children had serum and CSF acquired during the SE. Anti-neuronal antibodies were screened for in all children. These could not be detected in 13. One child (#5) showed a tissue staining of the rat brain hippocampus with CSF (Figure 1) but not with serum. Specificity was confirmed by showing neuronal surface staining of CSF using hippocampal embryonal primary neuron cultures (not shown). No known autoantibodies were detectable using cell-based assays. A follow-up CSF acquired 5 months after the onset of seizures did not show rat brain staining anymore. This child had a disease course atypical for FIRES in several respects and was, therefore, initially classified as atypical FIRES. He had a hypercortisolism due to pituitary insufficiency with ectopic neurohypophysis. The prodromal phase was very short with an onset of SE on day 1 of febrile illness. Additionally, an intrathecal IgM immunoglobulin synthesis was present on day 2 of the disease. Especially the presence of an intrathecal immunoglobulin synthesis in conjunction with the antibody findings triggered reclassification as AE. A second child (#12) was also classified as atypical FIRES. Status epilepticus was brief (less than a week) and responded to a methylprednisolone pulse. In the further course of the disease, acute worsening of the seizures occurred several times, which could be stabilized with methylprednisolone each time. No anti-neuronal or anti-glial antibodies were detected in this child.

In the course of the disease, one child died, five remained severely dependent on care and five regained some degree of independence but did develop a mild cognitive impairment. For three children, outcome was not reported. For every child for whom information on the outcome could be given, data were available at least 6 months after the onset of the disease. Two children died in the long-term of the disease, both initially developed a minimal responsive state.

The initial MRI and CT findings were normal in eight children. However, MRI changes appeared in seven children during the further acute phase. Three out of these seven, had unremarkable findings initially. The most common MRI findings during the acute phase were changes in the diffusion weighted imaging with changes in the hippocampal region in six children (Table 3). It remains unclear whether those results stem from the long SE or are specific to FIRES.

All 11 children with available information on treatment received immunosuppressive therapy, including plasmapheresis in one case. As far as documented, no child showed any immune defect in the performed immune function diagnostics. The extent of the diagnostics carried out and the available results varied greatly. Screenings for complement deficiency were performed in three children and screenings for rheumatic diseases in six children. Basic diagnostics for interleukin function and immunoglobulins were only documented for one child each. Another three children received genetic testing which did not identify an immunodeficiency.

The antiseizure medications included barbiturates, benzodiazepines, GABA derivatives, new antiseizure medications, carbonic anhydrase inhibitors, carboxamides, hydantoin derivatives, and hypnotics. Sedation and/or anesthesia was used in 10 of 11 children. A median of 10 different antiseizure medications/sedatives were used in each child (range 6–20 drugs). The deceased child was on 10 different antiseizure medications/sedatives. Barbiturates and benzodiazepines were used in all children. Levetiracetam was also used in all children but was stopped early in six children due to adverse effects. Eight children received a ketogenic diet, none with a clear clinical response.

## 4. Discussion

Febrile infection-related epilepsy syndrome is a rare but well-recognized pediatric epilepsy syndrome of unknown etiology. Because of phenotypic overlap with pediatric AE, FIRES has been speculated to be an encephalitis with yet unknown autoantibodies. Though, the absence of overt inflammatory changes of CSF and brain biopsies in typical FIRES (van Baalen et al., 2010) and the observed lack of response to immunotherapy/immunomodulatory therapy have argued against this. Using comprehensive autoantibody screening, including testing for MOG autoantibodies, we could confirm that underlying neuronal autoantibodies are not present in typical FIRES even if tested early during the disease. Otherwise, the one child in whom transient neuronal autoantibodies with unknown target could be observed, was one of two atypical cases of FIRES, characterized by a very short interval between febrile illness and the onset of seizures (1 day) and by mild inflammatory CSF changes. Consequently, he was reclassified as AE. His course of disease is similar to a recently reported FIRES-suspected case, which revealed GABA-A-R antibodies (Caputo et al., 2018).

We, therefore, propose a diagnostic workup for every patient with suspected FIRES, which includes the following: a standard diagnosis of neurotropic viruses, a minimal diagnosis of metabolic/endocrine abnormalities, a systematic testing for immunodeficiency, the concerns of intoxications (regardless of age, e.g., with a suspected Munchausen by proxy syndrome or accidental ingestion), the expansion of autoimmunity diagnostics to include the search for unknown antineuronal antibodies in the CSF, and the explicit search for neoplasms in every positive antibody proof and human genetic diagnostics.

A clear positive effect of a specific medication was not recognizable in our cohort. The use of coma therapy has not had a positive effect. Children with >8 antiseizure or sedative medications had a longer SE and more complications. This could be an indication that too aggressive status therapy may aggravate the outcome. In retrospect, only the passage of time seems to have led to a reduction in seizure activity.

Limitations of our prospective case series were the small amount of material, especially CSF (pediatric patients), making further antibody and cytokine studies difficult. Also, the quality of the clinical information on the individual course of the disease varied widely, as did the information about the initial laboratory CSF findings.

In conclusion, our prospective case series shows that typical cases of FIRES do not harbor neuronal or glial autoantibodies detectable with current techniques. Nevertheless, it demonstrates that patients with AE caused by unknown neuronal antibodies can be misclassified as atypical FIRES. Due to these findings, we infer that (1) all patients with suspected FIRES should undergo comprehensive autoantibody testing to detect underlying AE and initiate early immunotherapy and (2) atypical features of FIRES, e.g., early onset of SE during fever, should heighten the awareness of underlying AE even more. The underlying etiology of typical FIRES remains unclear.

## Data availability statement

The original contributions presented in the study are included in the article/supplementary material, further inquiries can be directed to the corresponding author.

## Ethics statement

The studies involving human participants were reviewed and approved by the ethics committee of the Faculty of Medicine at the Kiel University (CAU; D 414/21). Written informed consent to participate in this study was provided by the participants’ legal guardian/next of kin. Written informed consent was obtained from the minor(s)’ legal guardian/next of kin for the publication of any potentially identifiable images or data included in this article.

## Author contributions

JSW curated the data, conducted formal analyses and investigation, created visualization, developed the methodology, and wrote the original draft of the manuscript. K-PW acquired funding, developed the methodology, conducted investigation, provided resources, and reviewed and edited the manuscript. FL acquired funding, developed the methodology, conducted investigation, provided resources, validated the results, created visualization, and reviewed and edited the manuscript. GK conducted investigation and provided clinical data, provided resources, and reviewed and edited the manuscript. AvB conceptualized the project, conducted formal analysis, developed the methodology, oversaw project administration, supervised the study, validated the results, and reviewed and edited the manuscript. All authors contributed to the article and approved the submitted version.

## Funding

This work was in part supported by the German Federal Ministry of Science and Education (Comprehensive, Orchestrated, National Network to Explain, Categorize and Treat autoimmune encephalitis and allied diseases within the German NEtwork for Research on AuToimmune Encephalitis — CONNECT GENERATE; 01GM1908). This research did not receive any further specific grant from funding agencies in the public, commercial or not for profit sectors.

## Acknowledgments

We sincerely thank physicians, patients, and families for providing information and we thank all the staff at the institute for Clinical Chemistry at the University Hospital in Kiel/Lübeck, in particular Martina Jansen.

## Conflict of interest

The authors declare that the research was conducted in the absence of any commercial or financial relationships that could be construed as a potential conflict of interest.

## Publisher’s note

All claims expressed in this article are solely those of the authors and do not necessarily represent those of their affiliated organizations, or those of the publisher, the editors and the reviewers. Any product that may be evaluated in this article, or claim that may be made by its manufacturer, is not guaranteed or endorsed by the publisher.

## References

[ref1] Appenzeller S. Helbig I. Stephani U. Häusler M. Kluger G. Bungeroth M.. (2012). Febrile infection-related epilepsy syndrome (FIRES) is not caused by SCN1A, POLG, PCDH19 mutations or rare copy number variations. DMCN 54, 1144–1148. doi: 10.1111/j.1469-8749.2012.04435.x 23066759

[ref2] Armangue T. Leypoldt F. Dalmau J. (2014). Autoimmune encephalitis as differential diagnosis of infectious encephalitis. Curr. Opin. Neurol. 27, 361–368. doi: 10.1097/WCO.0000000000000087, PMID: 24792345 PMC4132825

[ref3] Armangue T. Olivé-Cirera G. Martínez-Hernandez E. Sepulveda M. Ruiz-Garcia R. Muñoz-Batista M.. (2020). Associations of paediatric demyelinating and encephalitic syndromes with myelin oligodendrocyte glycoprotein antibodies: a multicentre observational study. Lancet Neurol. 19, 234–246. doi: 10.1016/S1474-4422(19)30488-0, PMID: 32057303

[ref4] Banwell B. Bennett J. L. Marignier R. Kim H. J. Brilot F. Flanagan E. P.. (2023). Diagnosis of myelin oligodendrocyte glycoprotein antibody-associated disease: international MOGAD panel proposed criteria. Lancet Neurol. 22, 268–282. doi: 10.1016/S1474-4422(22)00431-8, PMID: 36706773

[ref5] Caputo D. Iorio R. Vigevano F. Fusco L. (2018). Febrile infection-related epilepsy syndrome (FIRES) with super-refractory status epilepticus revealing autoimmune encephalitis due to GABAAR antibodies. Eur. J. Paediatr. Neurol. 22, 182–185. doi: 10.1016/j.ejpn.2017.11.005, PMID: 29203057

[ref6] Caraballo R. H. Reyes G. Avaria M. F. L. Buompadre M. C. Gonzalez M. Fortini S.. (2013). Febrile infection-related epilepsy syndrome: a study of 12 patients. Seizure 22, 553–559. doi: 10.1016/j.seizure.2013.04.005, PMID: 23643626

[ref7] Dalmau J. Gleichman A. J. Hughes E. G. Rossi J. E. Peng X. Lai M.. (2008). Anti-NMDA-receptor encephalitis: case series and analysis of the effects of antibodies. Lancet Neurol. 7, 1091–1098. doi: 10.1016/S1474-4422(08)70224-2, PMID: 18851928 PMC2607118

[ref8] Dalmau J. Graus F. (2018). Antibody-mediated encephalitis. N. Engl. J. Med. 378, 840–851. doi: 10.1056/NEJMra1708712, PMID: 29490181

[ref9] Dalmau J. Lancaster E. Martinez-Hernandez E. Rosenfeld M. R. Balice-Gordon R. (2011). Clinical experience and laboratory investigations in patients with anti-NMDAR encephalitis. Lancet Neurol. 10, 63–74. doi: 10.1016/S1474-4422(10)70253-2, PMID: 21163445 PMC3158385

[ref10] Dalmau J. Vincent A. (2017). Do we need to measure specific antibodies in patients with limbic encephalitis? Neurology 88, 508–509. doi: 10.1212/WNL.0000000000003592, PMID: 28062718

[ref11] Gaspard N. Hirsch L. J. Sculier C. Loddenkemper T. van Baalen A. Lancrenon J.. (2018). New-onset refractory status epilepticus (NORSE) and febrile infection-related epilepsy syndrome (FIRES): state of the art and perspectives. Epilepsia 59, 745–752. doi: 10.1111/epi.14022, PMID: 29476535

[ref12] Lee H.-F. Chi C.-S. (2018). Febrile infection-related epilepsy syndrome (FIRES): therapeutic complications, long-term neurological and neuroimaging follow-up. Seizure 56, 53–59. doi: 10.1016/j.seizure.2018.02.003 29453111

[ref13] Leypoldt F. Armangue T. Dalmau J. (2015). Autoimmune encephalopathies. Ann. N. Y. Acad. Sci. 1338, 94–114. doi: 10.1111/nyas.12553, PMID: 25315420 PMC4363225

[ref14] Titulaer M. J. Höftberger R. Iizuka T. Leypoldt F. McCracken L. Cellucci T.. (2014). Overlapping demyelinating syndromes and anti–N-methyl-D-aspartate receptor encephalitis. Ann. Neurol. 75, 411–428. doi: 10.1002/ana.24117, PMID: 24700511 PMC4016175

[ref15] Udani V. Badheka R. Desai N. (2021). Prolonged fever: an atypical presentation in MOG antibody-associated disorders. Pediatr. Neurol. 122, 1–6. doi: 10.1016/j.pediatrneurol.2021.03.006, PMID: 34198219

[ref16] van Baalen A. Häusler M. Boor R. Rohr A. Sperner J. Kurlemann G.. (2010). Febrile infection-related epilepsy syndrome (FIRES): a nonencephalitic encephalopathy in childhood. Epilepsia 51, 1323–1328. doi: 10.1111/j.1528-1167.2010.02535.x, PMID: 20345937

[ref17] van Baalen A. Häusler M. Plecko-Startinig B. Strautmanis J. Vlaho S. Gebhardt B.. (2012). Febrile infection-related epilepsy syndrome without detectable autoantibodies and response to immunotherapy: a case series and discussion of epileptogenesis in FIRES. Neuropediatrics 43, 209–216. doi: 10.1055/s-0032-1323848, PMID: 22911482

[ref18] van Baalen A. Vezzani A. Häusler M. Kluger G. (2017). Febrile infection-related epilepsy syndrome: clinical review and hypotheses of epileptogenesis. Neuropediatrics 48, 5–18. doi: 10.1055/s-0036-1597271, PMID: 27919115

[ref19] Wandinger K.-P. Leypoldt F. Junker R. (2018). Autoantibody-mediated encephalitis. Dtsch. Arztebl. Int. 115, 666–673. doi: 10.3238/arztebl.2018.0666, PMID: 30381132 PMC6234470

